# Profiles of Volatile Biomarkers Detect Tuberculosis from Skin

**DOI:** 10.1002/advs.202100235

**Published:** 2021-06-02

**Authors:** Rotem Vishinkin, Rami Busool, Elias Mansour, Falk Fish, Ali Esmail, Parveen Kumar, Alaa Gharaa, John C. Cancilla, Jose S. Torrecilla, Girts Skenders, Marcis Leja, Keertan Dheda, Sarman Singh, Hossam Haick

**Affiliations:** ^1^ Department of Chemical Engineering and Russell Berrie Nanotechnology Institute Technion‐Israel Institute of Technology Haifa 3200003 Israel; ^2^ Centre for Lung Infection and Immunity Division of Pulmonology Department of Medicine and UCT Lung Institute & South African MRC/UCT Centre for the Study of Antimicrobial Resistance University of Cape Town Cape Town 7925 South Africa; ^3^ All India Institute of Medical Sciences New Delhi 110029 India; ^4^ The Scintillon Institute San Diego CA 92121 USA; ^5^ Department of Chemical and Materials Engineering Complutense University of Madrid Madrid 28040 Spain; ^6^ Institute of Clinical and Preventive Medicine University of Latvia and Riga east University Hospital Riga LV1079 Latvia; ^7^ Faculty of Infectious and Tropical Diseases Department of Infection Biology London School of Hygiene and Tropical Medicine London WC1E 7HT UK

**Keywords:** diagnosis, noninvasive approach, point‐of‐care test, sensor, skin, tuberculosis, wearable device

## Abstract

Tuberculosis (TB) is an infectious disease that threatens >10 million people annually. Despite advances in TB diagnostics, patients continue to receive an insufficient diagnosis as TB symptoms are not specific. Many existing biodiagnostic tests are slow, have low clinical performance, and can be unsuitable for resource‐limited settings. According to the World Health Organization (WHO), a rapid, sputum‐free, and cost‐effective triage test for real‐time detection of TB is urgently needed. This article reports on a new diagnostic pathway enabling a noninvasive, fast, and highly accurate way of detecting TB. The approach relies on TB‐specific volatile organic compounds (VOCs) that are detected and quantified from the skin headspace. A specifically designed nanomaterial‐based sensors array translates these findings into a point‐of‐care diagnosis by discriminating between active pulmonary TB patients and controls with sensitivity above 90%. This fulfills the WHO's triage test requirements and poses the potential to become a TB triage test.

## Graphical abstract

A noninvasive method for tuberculosis (TB) detection from skin is introduced in this paper. The method harnesses nanomaterial‐based sensors that are sensitive to patterns of volatile biomarkers that are linked with the illness. The results present TB‐related changes in the pattern with above 90% sensitivity detecting active TB patients with the sensors, demonstrating the potential to evolve into a new TB triage test.

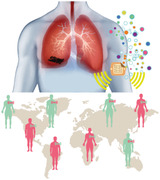

## 1 Introduction

Tuberculosis (TB) is a major health problem in the world.^[^ ^1^, ^2^ ^]^ Approximately 95% of TB cases occur in developing countries, including locations where people live on less than 1 USD per day. About one‐third of the world population has latent TB with a lifetime risk of 5 to 10% of developing active TB.^[^ ^3^ ^]^ HIV co‐infection, smoking, and malnutrition greatly increase this risk and speed up the TB epidemic.^[^ ^1^, ^2^ ^]^

TB is particularly difficult to diagnose in children and HIV co‐infected populations.^[^ ^4^ ^]^ Currently, around 3 million active TB cases are missed by the health systems worldwide.^[^ ^5^ ^]^ Despite advances in TB diagnostics, millions of patients continue to receive an incomplete or delayed diagnosis, as the physical signs and symptoms of TB are nonspecific.^[^ ^6^ ^]^ Many existing biodiagnostic tests are slow, have low sensitivity and/or specificity, and at times are too expensive or complex for resource‐limited settings. For example, a sputum smear (2.6 to 10.5 USD/examination, depending on the country) is too insensitive, and mycobacterial culture takes 4–8 weeks and at least 3 visits by the patient to finalize the diagnosis and begin treatment.^[^ ^7^ ^]^ This process is time‐consuming, labor‐intensive, requires highly trained technicians, and the method is based on challenging specimen collection and processing, both of which can greatly affect the sensitivity. Despite the high specificity, direct smear microscopy is relatively insensitive (20–80% sensitivity), since at least 5000 bacilli per milliliter of sputum are required for a positive result.^[^ ^8^ ^]^ The sensitivity is further reduced in patients with extra‐pulmonary TB, HIV‐compromised patients, and those with disease due to nontuberculous mycobacteria.^[^ ^6^, ^9^ ^]^ The limitations of using microscopy in low‐resource settings include poor‐quality reagents, unmaintained microscopes, and poor level of staff training. The only technology that can diagnose within 2 h, GeneXpert MTB/RIF, has a relatively limited sensitivity performance (88% as an initial test and 67% as an add‐on test for TB detection following a negative smear‐microscopy result).^[^ ^10^ ^]^ The equipment and consumables required are costly, as is the initial capital cost for the GeneXpert unit and additional costs for the delivery, installation, and service. The GeneXpert test is focused on sputum as the sample, and cannot differentiate between live and dead bacteria.^[^ ^11^ ^]^ Operating GeneXpert MTB/RIF requires a reliable energy source, security, and maintenance, and demands annual recalibration. Disadvantages of other methods include sputum as the processed sample, a requirement of a highly trained staff, high biosafety, and maintenance levels for performance, high cost. Therefore, new, and accurate TB diagnostic methods that can be produced and distributed at affordable prices for people living on <1 USD day^−1^ are critically needed.^[^ ^12^ ^]^ Early diagnosis and treatment initiation mitigate morbidity and disease spearing. From an economic point of view, TB caused about 12 billion USD to vanish from the global economy when considering the cost of TB patients' loss of productivity and death cases.^[^ ^13^ ^]^

We present an exploration and application of TB‐specific volatile organic compounds (VOCs) that can be detected from air trapped above the skin (the “skin headspace”). Deviation of these VOCs from the healthy VOC pattern in terms of their concentration range may indicate either TB infection or TB high infection risk. For translating these findings into a point‐of‐care reality, we present and discuss a new biomedical apparatus containing a flexible and wearable polymeric pouch for the collection and storage of skin VOCs and their analysis by nanomaterial‐based sensors array in conjunction with machine learning.^[^ ^14^, ^15^, ^16^ ^]^ The clinical offline study, involving such pouches, in two countries, shows that this approach provides fast, precise detection and classification of TB profiles from skin's headspace. Furthermore, as an additional step for realization toward a point of care diagnosis tool, an exploratory pilot study was conducted by applying a wearable electronic device directly on the skin of both healthy and active pulmonary TB patients.

## 2 Results

### 2.1 Study Design and Skin Sampling

**Figure 1:**
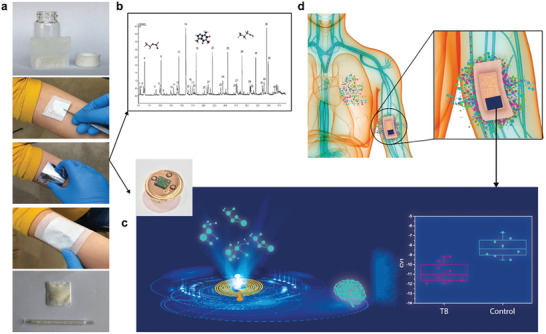
Study schematics. a) Skin headspace sampling procedure with poly(2,6‐diphenylphenylene oxide) polymer. The samples are transferred into glass tubes for two analyses: b) GC‐MS analysis of the collected samples; and c) nanomaterial‐based sensors array in conjugation machine learning analysis of the collected samples. d) A wearable device applied directly on the skin.

The study composed of three off‐line stages for examining the hypothesis and the science behind it and one demonstration stage with an online and *in‐situ* wearable device. The offline study took place through the inclusion of 636 subjects aged 22–60 years (**Figure** **1**a–c respectively): skin sampling, Gas Chromatography‐Mass Spectrometry (GC‐MS) analysis, and nanomaterial‐based sensors analysis in conjugation with machine learning methods. To create a robust tool for TB detection, samples and analysis were established in Cape Town in South Africa (*N* = 320) and in New Delhi in India (*N* = 316). The study population included newly diagnosed and confirmed pulmonary‐active TB cases, healthy volunteers, and confirmed non‐TB cases. Demographic and clinical data of the population is summarized in Tables S1 and S2 in the Supporting Information. 12 potential confounding factors, including HIV status and smoking habits, were monitored and assessed. Headspace samples from each participant were collected from the anterior arm area (inner arm) and chest area using : i) two offline porous polymeric pouches containing poly(2,6‐diphenylphenylene oxide) polymer, and ii) two polydimethylsiloxane (PDMS) sheets,^[^ ^17^ ^]^ covered by an adhesive medical tape. As a reference, poly(2,6‐diphenylphenylene oxide) pouches and PDMS sheets, for room sampling, were also included in the analysis in order to evaluate the exogenous impact. Comparative analysis has shown that sampling by poly(2,6‐diphenylphenylene oxide)‐based sampling at the anterior arm area, give the best and most stable results, making it the focus of our presentation in the current article. For more details about the various skin sampling, please see Section S2 in the Supporting Information.

### 2.2 Gas Chromatography Mass Spectrometry (GC‐MS) Analysis

In the second stage (Figure 1b), skin VOCs were qualitatively and quantitatively analyzed using GC‐MS with >80% nonzero values in all skin samples. Evaluation with GC‐MS cannot be used as a stand‐alone Point of Care (PoC) diagnostic tool. However, it provides preliminary proof for the feasibility of skin VOCs to serve as TB‐associated probe molecules. Furthermore, GC‐MS analysis can be used for investigation of the metabolic processes and their relation to VOCs,^[^ ^18^, ^19^ ^]^ and work up the nanomaterial‐based sensors and algorithms for better performance.^[^ ^15^, ^20^ ^]^

**Figure 2:**
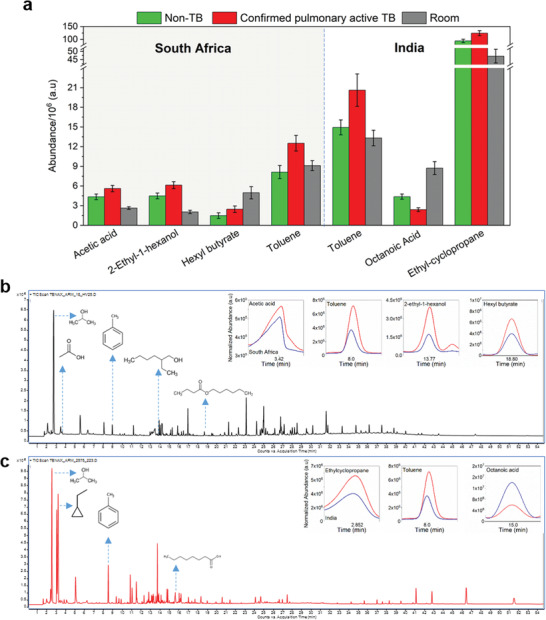
GC‐MS results. Samples from South Africa included 89 confirmed pulmonary active TB patients; 90 non‐TB patients with healthy controls; and 262 room samples.Samples from India included 89 confirmed pulmonary active TB patients; 193 non‐TB patients with healthy controls; and 193 room samples. a) An abundance of toluene, acetic acid, 2‐ethyl‐1‐hexanol, ethyl‐cyclopropane, hexyl butyrate, and octanoic acid among confirmed pulmonary active TB patients, non‐TB patients with healthy controls, and room samples, in both clinical sites. For hexyl butyrate, two extreme outlier points were excluded for the confirmed pulmonary active TB patients. b,c) Representative chromatograms with statistically significant VOCs and isopropyl alcohol (IPA) as a skin‐cleaning component, from South Africa and India, respectively. Inserts include representative chromatograms of relevant VOCs based on total ion count traces. Error bars represent standard errors. Steel method in comparison to the TB group as a posthoc testing *α* = 0.05 was used.

In South Africa, the analysis included: i) 89 confirmed pulmonary active TB patients; ii) 90 non‐TB patients with healthy controls; and iii) 262 room samples. Four VOCs were found to be significantly different in comparison to the pulmonary active TB group: toluene (retention time (R.T.) 8.4 min), acetic acid (R.T. 3.34 min), 2‐ethyl‐1‐hexanol (R.T. 13.7 min), and tentatively recognized hexyl butyrate (R.T. 18.8 min). In India, the analysis included: i) 89 confirmed pulmonary active TB patients; ii) 193 non‐TB patients with healthy controls; and iii) 193 room samples. Three VOCs were found to be significantly different in comparison to the pulmonary active TB, among them toluene, which was also at higher levels among confirmed active TB patients as in South Africa. Additional statistically significant VOCs included both tentatively recognized ethyl‐cyclopropane (R.T. 2.8 min) and octanoic acid (R.T. 15.23 min) compounds. **Figure** **2** and **Table** **1** present the information regarding each VOC in different test groups from both clinical sites. GC‐MS analysis of samples from both sites revealed TB‐associated skin VOC profiles that differed from those of the control profiles and room samples. In general, during disease formation, cells undergo structural and metabolic changes that change VOC patterns.^[^ ^18^, ^19^, ^21^ ^]^ As a result, some of these VOCs appear in distinctive compositions of the mixture, depending on whether a cell is healthy or infected.^[^ ^22^, ^23^, ^24^ ^]^

**Table 1:** Summary of VOCs’ properties, including simulated synthetic samples for validation and quantification of each VOC

	South African site				Clinical site in India		
	Acetic acid	2‐ethyl‐1‐hexanol	Hexyl butyrate	Toluene		Ethyl‐cyclopropane	Octanoic Acid
Formula	C_2_H_4_O_2_	C_8_H_18_O	C_10_H_20_O_2_	C_7_H_8_		C_5_H_10_	CH_3_(CH_2_)_6_CO_2_H
CAS no.	64‐19‐7	104‐76‐7	2639‐63‐6	108‐88‐3		1191‐96‐4	124‐07‐2
R.T. [min]	3.34	13.70	18.80	8.40		2.82	14.96
*m/z* [mass to charge]	43	57	43	91		42	60
Laboratory simulations: Mean ± s.e [ppb]							
Confirmed pulmonary active TB patients	936.47 ± 81.02	48.18 ± 4.09	‐	332.68 ± 30.49	539.02 ± 62.96	‐	‐
Non‐TB patients and healthy controls	722.63 ± 73.55	35.56 ± 3.33	‐	220.86 ± 25.60	394.30 ± 29.12	‐	‐
Room samples	432.32 ± 34.39	16.87 ± 1.90	‐	245.92 ± 19.19	353.20 ± 30.31	‐	‐
Lowest tested concentration [ppb]	700	6	‐	60		‐	‐
p‐value for subgroup comparisons^a)^							
Kruskal‐Wallis Test	<0.0001^b)^	<0.0001^b)^	<0.0001^b)^	0.0015^b)^	0.0003^c)^	<0.0001^c)^	<0.0001^c)^
Confirmed pulmonary active TB patients vs Non‐TB patients and healthy controls	0.0295	0.0313	0.0078	0.0022	0.0077	0.0093	0.0060 (0.0018)^d)^
Confirmed pulmonary active TB patients vs Room	<0.0001	<0.0001	0.0341	0.0048	<0.0001	<0.0001	<0.0001

^a)^ Post hoc testing with Steel method in comparison to the confirmed pulmonary active TB group as a *α* = 0.05;

^b)^ *α* = 0.00185;

^c)^ *α* = 0.0014;

^d)^ After elimination of two extreme points.

s.e = standard error.

Toluene was found at significantly higher levels among confirmed pulmonary active TB patients in both clinical sites, yielding similar ratios to non‐TB abundance, whereas the difference was negligible between the non‐TB and room groups. The studies in each of the two clinical sites were conducted within the same geographical areas, with a homogenous enrollment of the volunteers. Therefore, changes in toluene levels coming from confounding factors tend to be implausible. Toluene is an exogenous VOC associated with industrial pollution, as in the petrol industry.^[^ ^25^ ^]^ Therefore, it's abundance among room samples is not negligible. The degradation of toluene by *M. tuberculosis* strains is known to occur,^[^ ^26^ ^]^ as toluene is ubiquitous in the environment. In the human body, degradation occurs by cytochrome P450 isozymes in liver microsomes,^[^ ^27^, ^28^ ^]^ and the “normal” degradation rates depend on geographical differences.^[^ ^27^ ^]^ Furthermore, toluene emission from both breath and skin has already been reported.^[^ ^29^, ^30^, ^31^, ^32^ ^]^ Inhibitory action of toluene on the secretion of interferon‐gamma (IFN‐gamma), interleukin‐4 (IL‐4), and IL‐13 has been investigated in human peripheral blood mononuclear cells.^[^ ^33^ ^]^ These factors were associated with inhibition of autophagy during *M. tuberculosis* infection.^[^ ^34^ ^]^ Therefore, increased levels of toluene emission among confirmed active TB patients on both clinical sites suggests toluene's role both in bacterium metabolism and the immune system during the infection. In contrast to previously reported studies,^[^ ^35^, ^36^, ^37^, ^38^, ^39^, ^40^, ^41^ ^]^ which associated emission of toluene with poly(2,6‐diphenylphenylene oxide) (TENAX‐TA) degradation and storageour GC‐MS analysis of conditioned and unused poly(2,6‐diphenylphenylene oxide) pouches after 8 months storage at 4 °C refrigerator did not contain toluene residues. These findings strengthen toluene's accountability as a potential molecule as a probe for detecting TB. Other reported VOCs were present in skin headspace samples in both clinical sites; however, only a statistically significant difference between confirmed pulmonary active TB patients and non‐TB subjects was found at a single site. Variations in the study population, e.g., geographical location, cultural habits, genetics, and pollution levels, as well as food intake, may all be responsible for these variations. Higher levels of acetic acid among confirmed active TB patients may be evidence of the response of the immune system during infection.^[^ ^42^ ^]^ The low levels of this VOC in room samples in comparison to skin samples suggest an endogenous origin. Acetic acid is reported as toxic to *M. tuberculosis* due to its acid pH and strong bactericidal activity.^[^ ^42^ ^]^ Furthermore, acetic acid, as a part of some metabolic pathways, is emitted from both breath^[^ ^43^ ^]^ and skin^[^ ^44^, ^45^ ^]^ samples of healthy volunteers. 2‐ethyl‐1‐hexanol has been reported as a TB‐related VOC in the exhaled breath of patients, indicating its relevance to TB pathogenesis, strengthening the higher abundances among patients.^[^ ^46^ ^]^ 2‐ethyl‐1‐hexanol has also been reported as a VOC associated with cancer, being detected in both breath and urine.^[^ ^47^, ^48^, ^49^ ^]^ The presence of 2‐ethyl‐1‐hexanol in room samples is associated with microbial degradation of plasticizers in indoor air.^[^ ^50^ ^]^ Acetic acid and 2‐ethyl‐1‐hexanol VOCs were also found among the samples from India; however, there was no significant difference between confirmed pulmonary active TB patients and non‐TB subjects. The abundance of tentatively recognized hexyl butyrate was significantly higher at room samples in comparison to the skin samples and obtained the lowest abundance among the non‐TB group. The correlation to TB disease is unclear. Still, this compound is related to the lipid metabolism pathway and its derivatives were found in exhaled breath of healthy subjects.^[^ ^29^ ^]^ Though it is also known to have exogenous sources originating naturally from plants, and serves as a food additive,^[^ ^51^ ^]^ it can be found in cleaning and air‐care products.^[^ ^52^ ^]^

Increased levels of tentatively recognized ethyl‐cyclopropane.^[^ ^23^, ^53^, ^54^ ^]^ were observed among TB subjects at India's clinical site. Cyclopropanated‐mycolic acid is a common membrane lipid found in bacterial species, but only in a limited number of eukaryotes.^[^ ^55^, ^56^ ^]^ Though cyclopropanated mycolic acids are presumed to be important in TB pathogenesis, their specific role remains to be determined. Furthermore, the host innate immune activation is through cyclopropane modification of a glycolipid effector molecule.^[^ ^57^, ^58^ ^]^ As a hypothesis, the increased levels of cyclopropane among TB subjects emphasizes the critical key role of this compound in the infection progress. Octanoic acid (tentative recognition) was found with the highest abundance in room samples and with the lowest abundance among confirmed pulmonary active TB patients at the clinical site in India. This VOC is included here for the first time as a potential TB VOC biomarker. Octanoic acid has exogenous sources originated from industrial products, cleaning and flavoring agents, paints, and coatings, which may explain its high levels in indoor air samples.^[^ ^59^ ^]^ Lower levels among confirmed pulmonary active TB patients seem to be correlated to synthesis and deacylation of Ghrelin hormone, which is important to the regulation of body's energy and becomes damaged during TB disease – Ghrelin levels are higher among patients in comparison to control subjects.^[^ ^60^, ^61^ ^]^ Emission of octanoic acid has previously been reported via both breath and skin secretion pathways.^[^ ^29^ ^]^ It is important to accentuate that the validation and quantification of the compounds were carried out by calibration curves. The identification and quantification of ethyl‐cyclopropane, hexyl butyrate, and octanoic acid were not achieved due to permission system limitations in terms of operational temperatures needed to obtain a stable gas‐liquid equilibrium.

### 2.3 Nanomaterial‐Based Sensors Array Analysis

**Figure 3:**
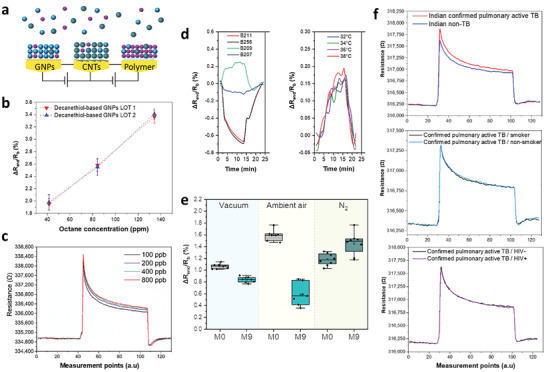
Sensor array responses. a) Schematic illustration a sensor array. b) Representative responses of decanethiol‐capped GNP sensors from two different batches toward increasing concentrations of octane. c) Representative response of the same sensor to increasing toluene concentration. d) Response rate to 0.6 ppb toluene in nitrogen of sensors based on different thiol ligands at 34 °C (left) and temperature effect on sensor response during decanethiol (B209)‐based GNPs exposure to toluene at 1.2 ppm (right). e) Representative responses of dodecanthiol‐based GNPs toward 1‐Methyl Naphthalene at 272 ppb in nitrogen exposure in different storage conditions at the starting point (M0) and after 9 months (M9). f) Representative signals of the same sensor to confirmed pulmonary active TB and non‐TB skin samples from the clinical site in India as well as confirmed pulmonary active TB with or without smoking habits and HIV infection.

In the third stage (Figure 1c), we designed an array of nanomaterial‐based sensors for detecting a variety of skin‐based TB VOCs.^[^ ^15^, ^16^, ^62^, ^63^ ^]^ TB is a complex condition involving many bodily systems, making it very difficult to be asscoaited with just one unique biomarker. For this reason, using a cross‐reactive approach in which a combination of nonselective sensors is used to provide one full picture or metabolic picture of the tested state can overcome the lack of specific markers.^[^ ^16^, ^64^, ^65^, ^66^, ^67^, ^68^ ^]^ In this approach, each sensor responds differently to individual or pattern of VOCs in the sample, aiding the evaluation of the VOC pattern in a qualitative and semi‐quantitative manner, while selectivity is achieved by predictive methods that are based on machine learning.^[^ ^15^, ^16^, ^20^, ^64^, ^65^, ^66^, ^69^, ^70^ ^]^ The sensors were based on chemiresistive films of spherical gold nanoparticles (GNPs; core diameter 3–4 nm) capped with different organic ligands, 2D random networks of single‐walled carbon nanotubes (RN‐SWCNTs) capped with different organic layers, and polymeric composites (**Figure** **3**a). Sensor response and reproducibility (within a batch and between batches) were evaluated upon exposure to octane at different concentrations (Figure 3b). The results exhibited high reproducability and repeatability. Prior to use with skin samples, the response of the sensors upon expoure to toluene, as a representative TB skin‐based probe molecule, was examined. The response of GNP‐based sensors was rapid, fully reversible, and responsive to a wide variety of toluene concentrations below and above levels reported above (Figure 3c). Different responses (positive and negative) were observed upon exposure to toluene at 0.6 ppb concentration in nitrogen at 34 °C (Figure 3d left). The effect of temperature on sensor response was evaluated during the expoure of decanethiol‐based GNPs to toluene at 1.2 ppm (Figure 3d right). No differences were observed between the responses over the tested range of temperatures. Dodecanthiol‐capped GNPs, as a representative example, were prepared and exposed to 1‐Methyl Naphthalene at 272 ppb in nitrogen, before and after 9 months storage period, at different storage conditions: vacuum (≈300 mbar), nitrogen (99.9998% pure) and room air: 18 °C, 40% RH. Figure 3e demonstrates the change in the resistivity (i.e., Δ*R*_end_/*R*_b_) for the different storage conditions. As can be seen, the change in the room air is the largest (35%), while the change in vacuum and nitrogen conditions is smaller, 19% and 17%, respectively. Moreover, the variances between the sensors' signals became larger in room air and nitrogen storage after 9 months of storage, in comparison to vacuum conditions. Therefore, sensors were selected to be stored in vacuumed conditions. Similar characteristics were obtained during sensor exposure to real skin headspace of healthy and TB subjects from both geographical locations with different confounding factors. Representative examples of the characteristics are shown in Figure 3f.

**Figure 4:**
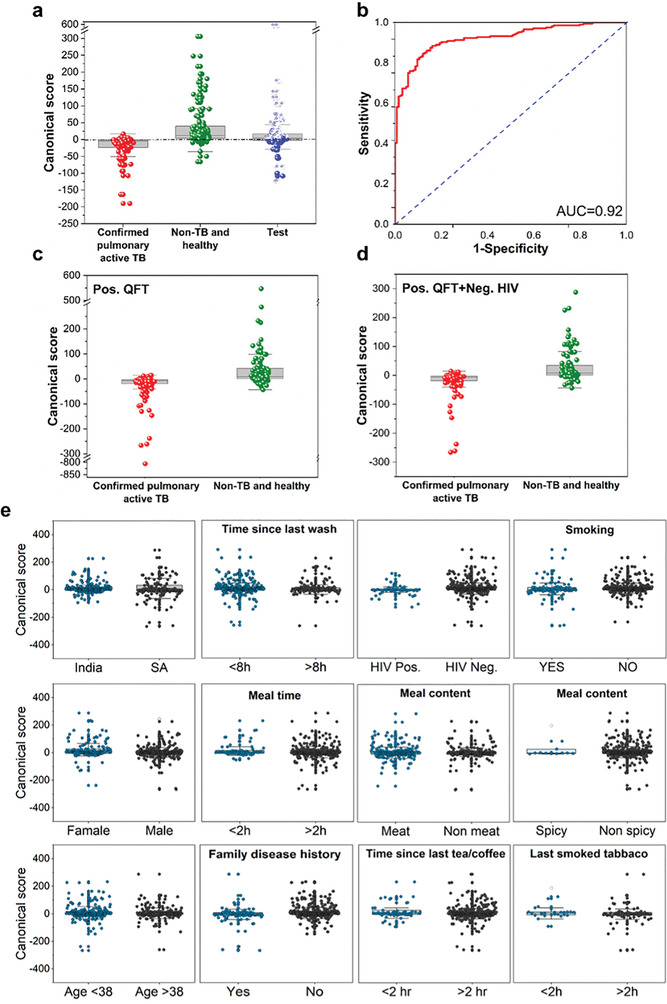
Quadratic DFA results of the global classifier. a) Boxplot of the canonical score. Each point represents one sample. The central dashed line represents Youden's cut‐point. Samples above the cut‐point are classified as non‐TB and healthy, and samples below it are classified as confirmed pulmonary active TB samples. Non‐TB and healthy samples of the test group are marked as open spheres, whereas confirmed pulmonary active TB samples of the test group are shown as closed spheres. b) Receiver operating characteristic (ROC) curve of the model. c) Boxplot of the canonical score for the subpopulation with QFT positive status. d) Boxplot of the canonical score for the subpopulation with QFT‐positive and HIV‐negative statuses. e) Boxplots of canonical score for confounding factors. QFT‐ QuantiFERON‐TB Gold test.

Use of an individual sensor of any of the chemistries (see the Experimental Section) gave a maximum accuracy of 64% for discriminating between the studied groups. To improve classification, signals from multiple sensors were combined, so that the information missed by one sensor is provided by the others, and the imprecision of a single sensor could be compensated by similar ones. Indeed, using multiple sensor signals together acts as an internal safety check on data – allowing the system software to discount temporarily erroneous readings, or, better still, correct them. Toward this end, Discriminate Factor Analysis (DFA) from 22 sensors (43 features) were used to separate confirmed pulmonary active TB patients from controls and non‐TB cases. The analysis was based on arm sampling and included a total of 475 samples, of which 176 were from confirmed pulmonary active TB patients and 299 from non‐TB and healthy volunteers. The evaluation of the performance of the quadratic DFA model was based on a randomly selected blinded‐test group (30% of the total dataset). In the training phase, the results yielded 84% accuracy, 90.3% sensitivity, and 80.3% specificity after training (**Figure** **4**a). The area under the curve of the receiver‐operating curve (ROC) scored 0.92 (Figure 4b). The analysis of the blinded‐test group (30%) resulted in 85.7% specificity, 90.4% sensitivity, and 87.4% accuracy. The performance in both phases meets the requirements of a triage test according to the WHO, a non‐sputum test with the performance of sensitivity of >90% and specificity a>70%.^[^ ^12^ ^]^ Based on the same model, further analysis targeting sub‐populations was carried out, which included discrimination between confirmed pulmonary active TB patients and non‐TB and healthy controls among (i) QuantiFERON‐TB Gold (QFT) positive population, and (ii) HIV negative within the positive QFT population. The clinical importance of this analysis is its ability to distinguish between confirmed active pulmonary TB and latent TB. Discrimination between confirmed pulmonary active TB and non‐TB and healthy controls among QFT positive population involved 301 samples; 131 confirmed pulmonary active TB cases and 170 non‐TB and healthy controls. Discrimination between confirmed pulmonary active TB and non‐TB and healthy controls among the QFT‐positive and the HIV‐negative population was based on 255 subjects; 96 confirmed pulmonary active TB cases and 159 non‐TB and healthy controls. The results of both analyses indicated similar performances, with 91.6–93.8% sensitivity, 70.4–71.2% specificity, and 78.4–81.1% accuracy (Figure 4c,d). 12 potential confounding factors and their influence on the results of the model were evaluated. These included gender, HIV status, time since the last bath, smoking status, and other factors. Their influence was based on the accuracy of the model used to discriminate between TB statuses. Figure 4e gives the accuracies, which were ≈50%, i.e., quite arbitrary. Thus, no significant difference within each of the confounding factors was found.

**Figure 5:**
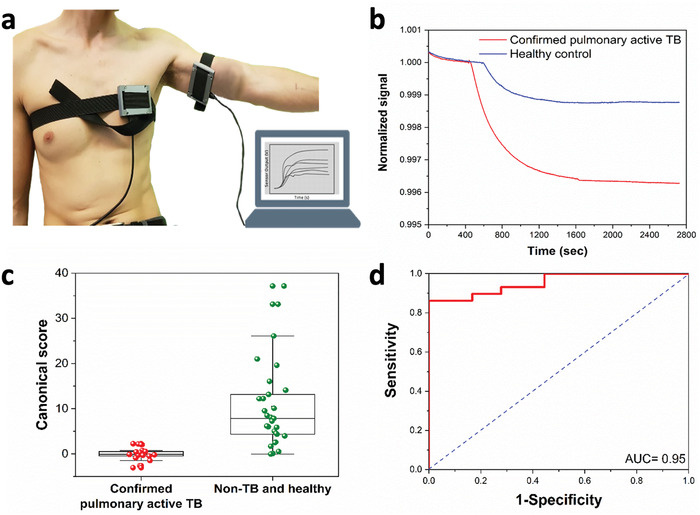
Wearable device TB diagnosis. a) Wearable devices on the chest and anterior arm of a volunteer. b) Representative normalized signals of one of the sensors in the wearable device attached to the anterior arm area. The plotted signal is the normalized resistance to the baseline resistance before a patch is attached to the experiment participant. c) Boxplot of the canonical score of linear DFA model. Each point represents one sample. d) ROC curve of the model. AUC = area under curve.

In the last stage (Figure 1d), an online exploratory pilot study was conducted by utilizing a wearable electronic device directly on the skin, without using absorbent material. The study was conducted in Riga, Latvia with a study cohort of 29 healthy subjects and 18 confirmed active pulmonary TB patients. The device, which included 8 nanomaterial‐based sensors, was placed on the chest and anterior part of the arm. Postprocessing analysus by quadratic DFA resulted in a leave‐one‐out validation model with 86.2% specificity, 94.4% sensitivity, and 89.4% accuracy in discriminating between active pulmonary TB patients and controls from sampling the anterior arm area (**Figure** **5**).

## 3 Discussion and Conclusions

Studies on the detection of TB VOCs have been previously reported from the headspace of TB cells,^[^ ^71^, ^72^, ^73^ ^]^ human exhaled breath,^[^ ^74^, ^75^, ^76^, ^77^ ^]^ and urine samples.^[^ ^78^ ^]^ However, to date the use of these techniques has been impeded by the compliance of the suspected subject, a need for moderately to highly expensive equipment, high levels of expertise required to operate the instruments, the speed required for sampling and analysis, and/or the requirement for pre‐concentration techniques.^[^ ^79^ ^]^ The GC‐MS analysis results presented herein provide the first evidence for a TB‐related VOC profile emitted into the skin headspace. Most of the introduced VOCs related to the TB are reported for the first time. Toluene is a shared VOC in both geographical locations, with similar proportions among the tested groups, identified and quantitated, and has a potential metabolic pathway related to TB. The degradation occurs in the human body normally by Cytochrome P450 isozymes in human liver microsomes^[^ ^27^, ^28^ ^]^ and the normal degradation rates may be subjected to geographical difference.^[^ ^27^ ^]^ Of all of that, toluene can be considered a probe molecule discriminating between different TB statuses.

The use of a cross‐reactive nanomaterial‐based sensors array in association with machine learning methods reveals the remarkable potential that, on one hand, creates a systematic screening for active case finding, and, on the other hand, rules out those people who do not present active pulmonary TB with high certainty. The array is exposed to the whole skin headspace sample, without selectivity toward specific VOCs.^[^ ^16^ ^]^ The selectivity toward a health status is achieved by machine learning models. The obtained results meet the WHO's target product profile criteria for a new TB triage test, expected to surpass 90% sensitivity and 70% specificity,^[^ ^12^ ^]^ without being affected by confounding factors, e.g. HIV status. VOC patterns are unique for every disease; therefore, the presence of one disease should not screen other diseases.^[^ ^15^ ^]^ As HIV is highly linked with TB infection, this allows one to eliminate possible cross‐effects in the diagnosis, as demonstrated in the TB disease classifier. Advanced discrimination among sub‐populations with both positive QFT and negative HIV statuses drew attention to the ability in distinguishing between confirmed active pulmonary TB and latent TB, or extra‐pulmonary TB disease in some rare cases. These results strengthen the potential of sensing TB‐related VOCs as a suitable method also to detect and diagnose latent cases, regardless of geographical differences. Future studies shall include an evaluation of the skin TB‐related VOCs for the detecting and monitoring treatment processes as well as inclusion of further geographic locations to form a global TB classifier.

The pilot study with the wearable device was a further step toward assimilation of the developed sensor‐based system to be applied in real‐time at healthcare facilities without the need for expensive laboratory equipment. Indeed, implementing the sensors array approach into an adhesive bandage is an additional step toward a simple and cost‐effective wearable sensing patch to establish a platform to address the TB epidemic risk in both developing and developed countries. This platform is expected to provide the foundation for the development of a wide variety of low‐end and high‐end wearable patches that can detect a wide variety of diseases and illnesses detectable by “sniffing” the corresponding skin‐emitted VOCs.^[^ ^14^, ^80^, ^81^, ^82^ ^]^

In summary, we developed a new method for detecting Tuberculosis from skin headspace, harnessing nanomaterial‐based sensors for noninvasive and easy sampling, utilizing volotalomics. This platform is suitable for serving as a triage test for Tuberculosis and can be easily modified to meet the needs for other diagnostics purposes.

## 4 Experimental Section

### Study Design

To ensure the stability of the rule‐in or rule‐out test in the proposed skin sampling, and based on a previous similar study,^[^ ^83^ ^]^ the sample sizes were determined by setting the lower limit of the Clopper–Pearson binomial confidence interval to be no more than 5% points below it. 210 per cohort (total) provides >90% power that the binomial 95% CI will be no wider than ±5.0% for sensitivity or specificity of 89%, or no wider than ±3.5% for sensitivity or specificity of 95%. In this multicentric study, absorbent skin patches for capturing the VOCs were developed at Technion, IIT, Israel. These patches were sent to 2 collaborating centers (All India Institute of Medical Sciences (AIIMS), New Delhi, India, and the Groote Schuur Hospital, Cape Town, South Africa) for VOC sampling from the study groups. During April 2016 and June 2017, samples were collected at AIIMS hospital in New Delhi. Sample collection took place in Groote Schuur Hospital in Cape Town from August 2015 until November 2016. All participants signed informed consent forms. The clinical trials received ethical approvals by the Ethical Committees of the respective hospitals: AIIMS, New Delhi: IEC/NP‐103/13.03.2015, RP‐39/2015 and University of Cape Town: 307/2014. The study design included three groups at each site with 105 participants per group: confirmed pulmonary active TB cases, healthy volunteers, and confirmed non‐TB cases. The clinical classification referred to 2 gold standards: sputum culture on liquid medium [BACTEC Mycobacteria Growth Indicator Tube MGIT 960 System (MGIT 960)] and GexeXpert MTB/RIF. Moreover, all the participants were screened for HIV and QuantiFERON‐TB Gold In‐Tube (QFT‐TB) tests for further evaluation on the effect of potential confounding factors. The participants were aged between 18–85 years and the following inclusion criteria were applied. Volunteers with a skin disease that was precluded at the sampling area were excluded from the studies. In addition, smoking within half an hour prior to testing was an additional exclusion criterion. The inclusion criteria for confirmed pulmonary active TB patients included: 1) clinical symptoms 2) positive microbiology (either a positive GeneXpert MTB/RIF or/and MGIT culture for M. tb; 3) newly diagnosed patients. For the non‐TB patients, the inclusion criteria included 1) clinical symptoms; 2) negative culture result (for HIV infected and uninfected) or Negative GeneXpert test result (HIV uninfected only); 3) chest x‐rays not supporting the diagnosis of active TB; 4) no clinical symptoms at follow‐up at 8 weeks. The inclusion criteria for healthy controls were: 1) no clinical symptoms in the past 12 months. For the clinical site in India, all the samples were collected at a single location with the same staff. For the SA site, the sampling was done in three clinics within Cape Town city with the same staff. The study cohort was designed initially for sampling with PDMS in both body locations and Tenax in the chest area only. The decision to include Tenax sampling in the anterior arm area was made after the beginning of the sampling in the two clinical sites. Therefore, the number of Tenax‐based samples from an anterior arm area was lower than other sampling procedures. In addition, before statistical analysis, due to technical reasons such as broken vials during shipment, or during GC‐MS failures, samples were excluded. For the exploratory study for the wearable device, similar inclusion and exclusion criteria were applied in Riga, Latvia (Nr.12‐A/19). The study included 18 confirmed pulmonary active TB patients and 29 healthy controls.

### Preparation of Poly(2,6‐diphenylphenylene oxide)‐Based Pouch as Off‐Line Sampling Tool

132 mg of 20/35 meshed poly(2,6‐diphenylphenylene oxide) (Buchem BV) was used as an absorbing material. Poly(2,6‐diphenylphenylene oxide) was thermally conditioned at 300 °C in a constant flow of pure nitrogen for 180 min. The polymer was conditioned in a glass tube with a conditioned glass wool (Sigma‐Aldrich) at 240 °C for 48 h. After conditioning it was stored in polyester meshed pouches (40.3 mm X 65.11 mm, mesh opening: 47µ) (SAATI), which had been cleaned with a solution of 5% Decon 90 decontaminant (Decon Laboratories) in distilled water (18.2 MΩ) and later stored in a vacuum oven at 100 °C for >15 h. The absorbing materials were stored in vials, closed, and wrapped with Parafilm at an average temperature of 4 °C. the shelf‐life examination was done at 4 °C storage conditions for up to 8 months.

### Sampling Procedure

Each participant wore two poly(2,6‐diphenylphenylene oxide) pouches on an anterior arm area, which had to be analyzed by both GC/MS and a nanomaterial‐based sensor array. In addition, room samples were collected for each participant to monitor the exogenous VOCs during skin sampling. The absorbent materials were placed on the skin after cleaning with a sterile alcohol pad (saturated with >70% isopropyl alcohol) for 10 min before sampling. The used pads were discarded into a closed bag, in order to reduce the IPA vapors in the sampling room. The absorbent materials were covered with aluminum foil and sealed with medical adhesive tape to avoid any VOCs absorption from the surrounding environment. No shaving procedure was done in order to avoid injury to the skin and change the VOC pattern. Sampling was done over 1h. A questionnaire was filled for every participant and the absorbent material vial numbers were also documented. The questionnaire included data regarding the main content of the last food and drink taken prior to sampling, hygiene, vaccinations, genetic, chronic, and infectious diseases, family TB history, smoking and drinking habits, allergies, medications and vitamins, among other details. For the room samples, the poly(2,6‐diphenylphenylene oxide) pouch was placed on the table near the participant for 1h to be exposed to the room air. During the whole sampling process, participants wore facemasks. After sampling, the absorbing materials were stored in vials, closed, and wrapped with Parafilm. These samples were stored in a refrigerator atan average temperature of 4 °C up to a maximum period time of 8 months. The air transportation of the samples was with the same conditions. Opening of the vials was done in a biological hood in a BSL2+ laboratory with the needed protective equipment. The disinfecting material was Oosafe Surface Disinfectant (SparMED) which does not contain alcohol and bactericide (confirmed for M. tuberculosis). The manufacturer claims that this disinfectant does not release VOCs. Prior to the instrumental runs of the samples, the polyester pouches were cut, and the poly(2,6‐diphenylphenylene oxide) powder was transferred immediately into a glass tube containing a glass wool stopper. After the transfer, the second opening was closed manually with the glass wool and capped from both sides.

### Sample Analysis with the Gas Chromatography–Mass Spectrometry (GC‐MS)

An analytical evaluation of the compounds absorbed on the poly(2,6‐diphenylphenylene oxide) was done with a GC/MS‐QP2010 instrument (Shimadzu Corporation). It was equipped with an SLB‐5ms capillary column (with 5% phenyl methyl siloxane; 30 meters in length; 0.25 mm internal diameter; 0.5 mm thicknesses; purchased from Sigma‐Aldrich), and was combined with a thermal desorption (TD) system (TD20; Shimadzu Corporation). Samples were analyzed by the GC‐system in split mode (20%) at 30 cm sec^−1^ constant linear speed and in a 0.70 ml min^−1^ column flow. The following oven temperature profile was set: (a) 6 min at 40 °C; (b) 13 °C min^−1^ ramp up until 170 °C; (c) a hold‐time 2 min; (d) 6 °C min^−1^ ramp up until 300 °C; and (e) 15 min at 300 °C. The run duration was 55 min in total. A mixture of alkane standard solution C_8_‐C_20_ in hexane solvent (Sigma‐Aldrich) was used as an external standard for GC/MS system calibration and normalization of the retention times and abundance changes as a result of column aging. Compounds present in >80% of skin samples until R.T. 30 min, were included in the analysis, as after 30 min compound release from Tenax and glass wool components occurred. Sample chromatograms were further analyzed using an open‐source program OpenChrom Community Edition, version 1.1, and custom codes using MATLAB version 9.5.0.944444 (R2018b). The chromatograms were converted into txt files with the following batch processing: 1. Denoising filter (*M.Z*. 73, 75, 28, 147, 207, 221, 281, 295, 335, 429); 2. Savitzky‐Golay filter; 3. Smoothed TIC baseline detector; 4. Peak detector first derivative (MSD); and 5. Combined integrator trapezoid. The analysis steps were programmed in order to overcome retention time shifts due to a prolonged study run and changes in the detector sensitivity.

### Calibration Curves of VOCs for GC‐MS

Identification and quantification of the VOCs that were found to be significant, involved creation of a calibration curve for each candidate. VOCs at different concentrations were generated using a commercial permeation/diffusion tube dilution (PDTD) system (Umwelttechnik MCZ, Germany). The system allows controlling the concentration of the VOCs. Purified dry nitrogen (99.9999%) from a commercial nitrogen generator (N‐30, On Site Gas Systems, USA) equipped with a nitrogen purifier was used as a carrier gas. Samples were actively absorbed on poly(2,6‐diphenylphenylene oxide) tubes at the same weight (132 mg) as used for the skin sampling from the PDTD system by pumping for 2.5 min at a flow rate of 0.2 L min^−1^. 3–5 repetitions were done per concentration. The following concentrations were generated: toluene: 60100212300400583744 ppb; acetic acid: 700, 900, 1100, 1300 ppb and for 2‐ethyl‐1‐hexanol: 6, 20, 40, 60, 80 ppb. The samples were analyzed by the same GC/MS method, and a calibration curve was generated and compared to the abundance range of the clinical and room samples, using a weighted linear regression with errors in abundance. A mixture of alkane standard solution C_8_‐C_20_ in hexane solvent was used during the calibration as well.

### Sample Analysis with the Nanomaterial‐Based Sensor Array

A stainless‐steel cell for exposure contained an array of 40 nanomaterial‐based sensors mounted on a customized polytetrafluoroethene circuit. The sensors included gold‐nanoparticles (organically stabilized spherical Au nanoparticles (core diameter: 3–4 nm), 2D random networks of single‐walled carbon nanotubes (RN‐SWCNTs), and polymers capped with different organic layers. For the modeling, the following sensors proved to be key: (i) Au nanoparticles covered with octadecanethiol, decanethiol, tert‐dodecanethiol, butanethiol, 2‐ethylhexanethiol, dibutyl disulfide, 4‐chlorobenzenemethanethiol, 3‐ethoxythiophenol, octadecylamine (Sigma‐Aldrich) and 2‐nitro‐4‐(trifluoromethyl) benzenethiol, benzylmercaptan (Carbone scientific). (ii) Random networks (RNs) of carbon nanotubes (CNTs) with crystal hexa‐perihexabenzocoronene (HBC) with C12 chemiresistor (HBC‐C12). (iii) Polymer composites black carbon with poly(propylene‐urethaneureaphenyl‐disulfide) PPUU‐2S chemiresistor and a composite of black carbon with poly(propylene‐urethaneureaphenyl‐disulfide) PPUU‐2S mixed with poly(urethane‐carboxyphenyl‐disulfide) PUC‐2S chemiresistor. Details regarding the fabrication and modification of the abovementioned sensors can be found in the literature.^[^ ^63^, ^84^, ^85^, ^86^ ^]^ The poly(2,6‐diphenylphenylene oxide) samples were transferred prior to analysis into empty thermal desorption (TD) tubes (Sigma‐Aldrich), containing glass wool stoppers, compatible with the TD system. The samples were thermally desorbed at 270 °C for 10 min in an auto‐sampler desorption system (TD20; Shimadzu Corporation, Japan). The sample was injected into the GC‐system (Shimadzu Corporation, Japan) in a direct (splitless) mode at a constant 3 mL min^−1^ total flow and the desorbed sample was temporarily stored in a stainless‐steel column (150 °C). The samples from the TD were then delivered by a 6‐way Valco valve, equipped with 10 mL stainless steel loop (VICI, Valco Instruments Company Inc., USA) into a stainless‐steel chamber containing the sensors with a volume of 330 cm^3^. When a one‐way valve connecting the chamber to the column was open, the sample was sucked into the chamber, while the remaining volume was filled with N2 until reaching atmospheric pressure in the chamber. A Keithley 2701 DMM data acquisition/data‐logging system was used to measure the resistance of all the sensors simultaneously as a function of time. The sensors’ baseline responses were recorded for 5 min in a vacuum (≈30 mTorr), 5 min under pure nitrogen (99.999%), 5 min in a vacuum, and 5 min under the sample exposure, followed by a further 3 min under vacuum conditions. To supervise the sensor's functionality during the experiment, and to overcome possible sensor response drift, a fixed calibration gas mixture containing 11.5 ppm isopropyl alcohol, 2.8 ppm mesitylene and 0.6 ppm 2‐ethyl‐1‐hexanol was exposed to the sensors daily. This calibration gas was generated PDTD system. The calibration mixture was absorbed on a clean tube for 2 min. Several features are extracted from each of the sensor's signals upon exposure, including area under the curve, delta R peak, delta R middle, and delta R end. The last three features are based on the difference between the baseline resistance, usually during vacuum, and the resistance during the response toward the exposure: peak point, middle part, and the end part of the signal.

### Discriminant Function Analysis (DFA)

DFA is a statistical method for data analysis when the groups to be discriminated are defined (labeled) before analysis.^[^ ^87^ ^]^ The input variables are the features extracted from sensors’ responses toward the skin samples. The decision on either linear or quadratic model was based on the homogeneity of the variance‐covariance matrices of the tested groups according to statistical tests, e.g. Bartlett's.^[^ ^88^, ^89^ ^]^ During this study, equal prior probabilities were set to confirm pulmonary active TB and non‐TB with healthy volunteers, respectively. For the off‐line approach, the model was evaluated by randomly splitting the original database into 70% training set and 30% test set. The number of the features used was examined for preventing the potential overfitting by following the accuracies of both training and test datasets as a function of the number of features. In this dataset, a model with >48 features was close to the overfitting range, whereas the proposed model contains fewer features (Figure S7, Supporting Information). For the on‐line device system, a quadratic DFA model was applied, based on 3 sensor features with leave‐one‐out validation, due to the low number of samples. Potential overfitting was prevented by limiting the number of the model features to only 4, leading to a ratio of 1:11 between the sample number and the model features. Feature selection and related calculations relied on Python version 3.7.

### Wearable Device for Online Measuring

The device, connected to the PC, and the developed software was launched. Initially, the device samples room air for 10 min, then the device was strapped to the participant for 60 min. There was no direct contact between the skin and the sensors. The device included eight sensors based on capped gold nanoparticles as described in previous method sections. On exposure, several features, such as area under the curve, delta R peak, delta R middle, and delta R end, are extracted from the sensor signal. The last three characteristics are based on the difference between the resistance of the baseline, typically during the room air exposure, and the resistance upon exposure response: peak point, middle, and end part, respectively. The obtained quadratic DFA model was based on three features from three sensors.

### Statistical Analysis

GC‐MS analysis in South Africa included i) 89 confirmed pulmonary active TB patients; ii) 90 non‐TB patients with healthy controls; and iii) 262 room samples (a total of 441 samples). In India, the analysis was based on i) 89 confirmed pulmonary active TB patients; ii) 193 non‐TB patients with healthy controls; and iii) 193 room samples (a total of 475 samples). The data pre‐processing included normalization of the peak abundances with an external standard for GC/MS system calibration. No transformation or outlier elimination was done except for ethyl‐cyclopropane analysis; 60 samples were excluded due to saturated peak of IPA, leading to the following tested groups: i) 85 confirmed pulmonary active TB patients; ii) 182 non‐TB patients with healthy controls; and iii) 148 room samples.

Statistical evaluation was based on an adjusted p‐value for multiple peaks, using a nonparametric Kruskal‐Wallis test and a nonparametric Steel method (0.05) in comparison to the TB group as posthoc testing. For the nonparametric Kruskal‐Wallis test, the following P values of 0.00185 and 0.00143 were used, respectively, for the clinical sites in South Africa and India. GC‐MS analysis revealed 27 and 35 peaks with at least 80% non‐zero values in all skin samples collected fron the clinical sites in South Africa and India, respectively, after discarding column bleeding compounds, such as siloxane‐related ones. All the peaks presented a right‐skewed distribution. All quantitative data were given as mean ± standard error. JMP software version 14.0.0 (SAS Institute Inc., Cary, NC, USA, 1989–2005) was used for statistical evaluation.

## Conflict of Interest

The authors declare no conflict of interest.

## Supporting information

Supporting Information

## Data Availability

The data that support the findings of this study are available from the corresponding author upon reasonable request.
