# Supplementary material for: Profiles of Volatile Biomarkers Detect Tuberculosis from Skin
Source: Adv Sci (Weinh). 2021 Jun 2;8(15):2100235. doi: 10.1002/advs.202100235 (PMC8336503; doi:10.1002/advs.202100235)
Supplement: Supplementary file 1 — Supporting Information [file ADVS-8-2100235-s001.pdf]

© 2021 Wiley-VCH GmbH

ADVANCED SCIENCE Open Access

Supporting Information

for *Adv. Sci.,* DOI: 10.1002/advs.202100235

Profiles of Volatile Biomarkers Detect Tuberculosis from Skin

*Rotem Vishinkin, Rami Busool, Elias Mansour, Falk Fish, Ali Esmail, Parveen Kumar, Alaa Gharaa, John C. Cancilla, Jose S. Torrecilla, Girts Skenders, Marcis Leja, Keertan Dheda, Sarman Singh and Hossam Haick**

Supporting Information:

Profiles of Volatile Biomarkers Detect Tuberculosis from Skin

*Rotem Vishinkin, Rami Busool, Elias Mansour, Falk Fish, Ali Esmail, Parveen Kumar, Alaa Gharaa, John C. Cancilla, Jose S. Torrecilla, Girts Skenders, Marcis Leja, Keertan Dheda, Sarman Singh and Hossam Haick**

Dr. R. Vishinkin, R. Busool, E. Mansour, Dr. Falk Fish, A. Gharaa, Prof. H. Haick
Department of Chemical Engineering and Russell Berrie Nanotechnology Institute
Technion-Israel Institute of Technology
Haifa 3200003, Israel
Email: hhossam@technion.ac.il

Dr. A. Esmail, Prof. K. Dheda
Centre for Lung Infection and Immunity, Division of Pulmonology, Department of Medicine and UCT Lung Institute & South African MRC/UCT Centre for the Study of Antimicrobial Resistance
University of Cape Town
Cape Town 7925, South Africa

Prof. K. Dheda
Faculty of Infectious and Tropical Diseases, Department of Infection Biology
London School of Hygiene and Tropical Medicine
London WC1E 7HT, UK

Dr. P. Kumar, Prof. S. Singh
All India Institute of Medical Sciences
New Delhi 110029, India

Dr. J. C. Cancilla
The Scintillon Institute
San Diego 92121, United States

Prof. J. S. Torrecilla
Department of Chemical and Materials Engineering
Complutense University of Madrid
Madrid 28040, Spain

Dr. G. Skenders, Prof. M. Leja
Institute of Clinical and Preventive Medicine
University of Latvia and Riga east University Hospital
Riga LV1079, Latvia

1. Characterization of the Studied Populations

Table S1. South African population characterization summary.

	Confirmed Pulmonary Active TB Patients	Non-TB Patients	Healthy Control
**Number of patients**	109	105	106
**Female: Male ratio**	35 : 74	43 : 62	84 : 22
**Age [years]**	38.54 ± 12.03	40.35 ± 12.58	37.0 ± 10.09
**Born in South Africa [%]**	100%	99%	93%
**Smoking status: Non-smokers: Smokers**	59 : 50	43 : 62	86 : 20
**Smoking habits** (1) 1-5 times/day ; (2) 6-10 times/day; (3) 10-15 times/day; (4) 16-20 times/day ; (5) > 20 times/day	(1) 23 : (2) 20 : (3) 3 : (4) 1	(1) 26 : (2) 20 : (3) 8 : (4) 2 : (5) 2	(1) 10 : (2) 7 : (3) 2 : (4) 1
**HIV status Positive: Negative**	55 : 54	36 : 69	18 : 88
**Time since last wash [hr]**	6.24 ± 6.76	8.028 ± 8.589	4.865 ± 3.29
**Time since last meal [hr]**	10:91 ± 7.79	12.815 ± 8.08	7.42 ± 6.82
**Main contain of last meal**	54 Meat, 30 sweet food, 18 vegetables, 7 other	66 Meat, 12 sweet food, 7 vegetables, 20 other	48 Meat, 23 sweet food, 25 vegetables, 10 other
**TB family history**	23	21	14
**QFT status Pos. : Neg. : IND**	88 : 14 : 14	64 : 36 : 5	69 : 35 : 2
**GeneXpert result**	103 Pos.: 3 Neg.	105 Neg.	n/a
**Culture result**	103 Pos. :4 Neg. :2 IND	105 Neg.	n/a

**Previous TB history**	25	3	13
**Last alcohol consumption prior sampling**<br>(1) No alcohol consumption; (2) 2-5 hours; (3) day before; (4) week before; (5) > 2 weeks before	(1) 70 : (2) 1 : (3) 6 : (4) 19: (5) 7	(1) 75 : (3) 15 : (4) 12: (5) 2	(1) 76 : (2) 1 : (3) 12 : (4) 11: (5) 7
**Chronic diseases (excluding HIV)**	1 Asthma, 1 Diabetes	2 Asthma, 1 hypertension, 1 epilepsy	3 Asthma
**(2,6-diphenyl-p-phenyleneoxide)-based anterior arm headspace area samples**	92	10	91

Mean ± standard deviation, QFT = QuantiFERON-TB Gold test, IND - Indeterminate

Table S2. Indian population characterization summary.

	Confirmed Pulmonary Active TB Patients	Non-TB Patients	Healthy Control
**Number of patients**	107	103	106
**Female : Male ratio**	28 : 79	27 : 76	26 : 80
**Age [years]**	41.57 ± 17.34	42.37 ±17.06	31.88 ± 8.93
**Born in India [%]**	100%	100%	100%
**Smoking status** **Non-smokers: Smokers**	96: 11	86: 17	85: 21
**Smoking habits** (1) 1-5 times/day; (2) 6-10 times/day; (3) 10-15 times/day; (4) 16-20 times/day; (5) > 20 times/day	(1) 8 : (2) 2 : (3) 1	(1) 11 : (2) 3 : (3) 2 : (5) 1	(1) 15 : (2) 2 : (3) 2 : (4) 1 : (5) 1
**HIV status Positive: Negative**	106 Neg. :1 IND	103 Neg.	106 Neg.
**Time since last wash [hr]**	25.72 ± 70.6	17.19 ± 21.47	5.83 ± 6.38
**Time since last** [**hr]**	10.89 ± 8.34	11.92 ± 8.02	4.52 ± 5.02
**Main contain of last meal**	68 meat, 36 vegetables, 3 other	68 meat, 24 vegetables, 11 other	59 meat, 46 vegetables, 1 other
**TB family history**	18	18	9
**QFT status Pos. : Neg. : IND**	74 : 32 : 1	45 : 58 : 0	61 : 44 : 1
**GeneXpert result**	92 Pos.: 15 Neg.	103 Neg.	n/a
**Culture result**	97 Pos.: 10 Neg.	103 Neg.	n/a
**Previous TB history**	26	29	6
**Last alcohol consumption prior sampling** (1) No alcohol consumption; (2) 1-5 hours; (3) day before; (4) week before; (5) > 2 weeks before	(1) 99 : (2) 1 : (3) 1 : (5) 6	(1) 94 : (2) 1 : (4) 5: (5) 3	(1) 81 : (2) 1 : (3) 4 : (4) 13: (5) 7

**Chronic diseases**	3Asthma , 2 COPD, 2 Bronchitis, 1 Aspergillosis, 1 Giardiasis	12 Asthma, 9 COPD, 4 Bronchitis, 1 Cardiomyopathy	n/a
**(2,6-diphenyl-p-phenyleneoxide)-based anterior arm headspace area samples**	90	92	106

Mean ± standard deviation, QFT = QuantiFERON-TB Gold test, IND - Indeterminate

2. Off-line Tools for Collecting VOCs from Skin

In the literature review on the collection of skin VOCs, there are many different methods, usually involving uncomfortable sampling procedures, *e.g.*, wrapping the desired area with a plastic bag. During the experiments, comfortable sampling methods that will increase the volunteer's compliance were chosen. Two different absorbing materials were investigated and characterized. Protocols for fabrication, sampling, and storage for both materials were established.

2.1 Characterization of PDMS as a Sampling Tool

Several important parameters related to the Polydimethylsiloxane (PDMS) (Specialty Silicone Products Inc., USA) were investigated and optimized in order to fit as a sampling tool to detection of TB VOCs from the skin:

1. PDMS Dimensions optimized to 2.5 cm X 0.5 cm with 0.017" thickness.
2. PDMS cleaning process was evaluated in a serial experiment using: 1) Decon 90 with distilled water; 2) acetone and methanol washing; and 3) Plasma process. The optimal cleaning process was Decon 90 with distilled water.
3. Thermal conditioning process was examined in a range of temperatures from 180°C to 270°C, under a constant pure gas flow (Nitrogen or Helium) for up to 90 minutes. The optimal conditioning temperature was determined as 270°C, under a constant flow of Nitrogen for 60 minutes. An example of PDMS before and after thermal conditioning is showed in **Figure S1**. The abundance of the materials was significantly reduced after the conditioning process. The mutual materials between the two graphs are mostly silicon produces from the PDMS and Gas chromatography–mass spectrometry (GC-MS) column.

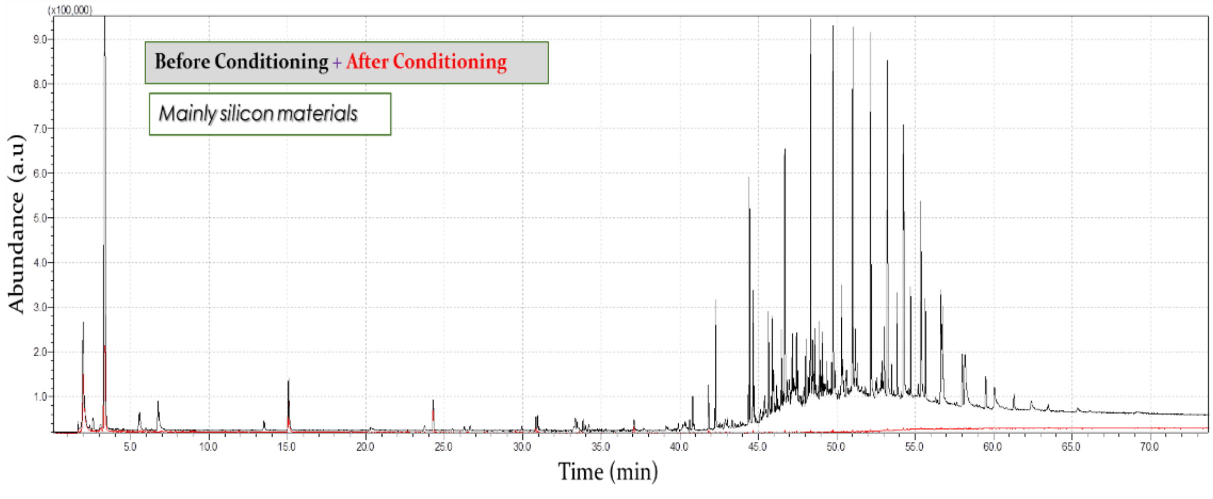

Figure S1. GC MS chromatography for PDMS before the chosen conditioning process (black) and after conditioning process (red).

4. Shelf life of the conditioned PDMS sheets was also examined at 4°C storage conditions. It was found that the samples in the glass vials, sealed with parafilm be stored up to 8 months.
5. Cover material/attachment procedure of PDMS to skin was tested to reduce the background noise during the sampling from both the cover itself and the environment. In the literature the cover material was a gauze pad[14]; however, during our evaluation very noisy results were received. A series of experiments was held in which several different cover materials were tested including gauze pad, parafilm, and aluminum foil (*see* **Figure S2**). A room sample was collected by hanging the PDMS in the room for the same duration of the experiment; the results are shown as a blue chromatogram in a case of aluminum foil (**Figure S2c**). The marked areas in cases of gauze pad and Parafilm (**Figure S2b**) highlight the high levels of the noise caused by the cover materials as the black chromatogram had the same abundance (or even higher) as the materials from the skin test. In the case of aluminum foil as a cover material, the noise levels are substantially negligible compared to the skin test. This indicates that the VOCs found in the skin sample are indeed VOCs emitted from the skin. As another control test, the room's PDMS sample had a negligible VOC profile in comparison to the skin sample, indicating that an efficient cover that protects the skin sampling from air pollutants.

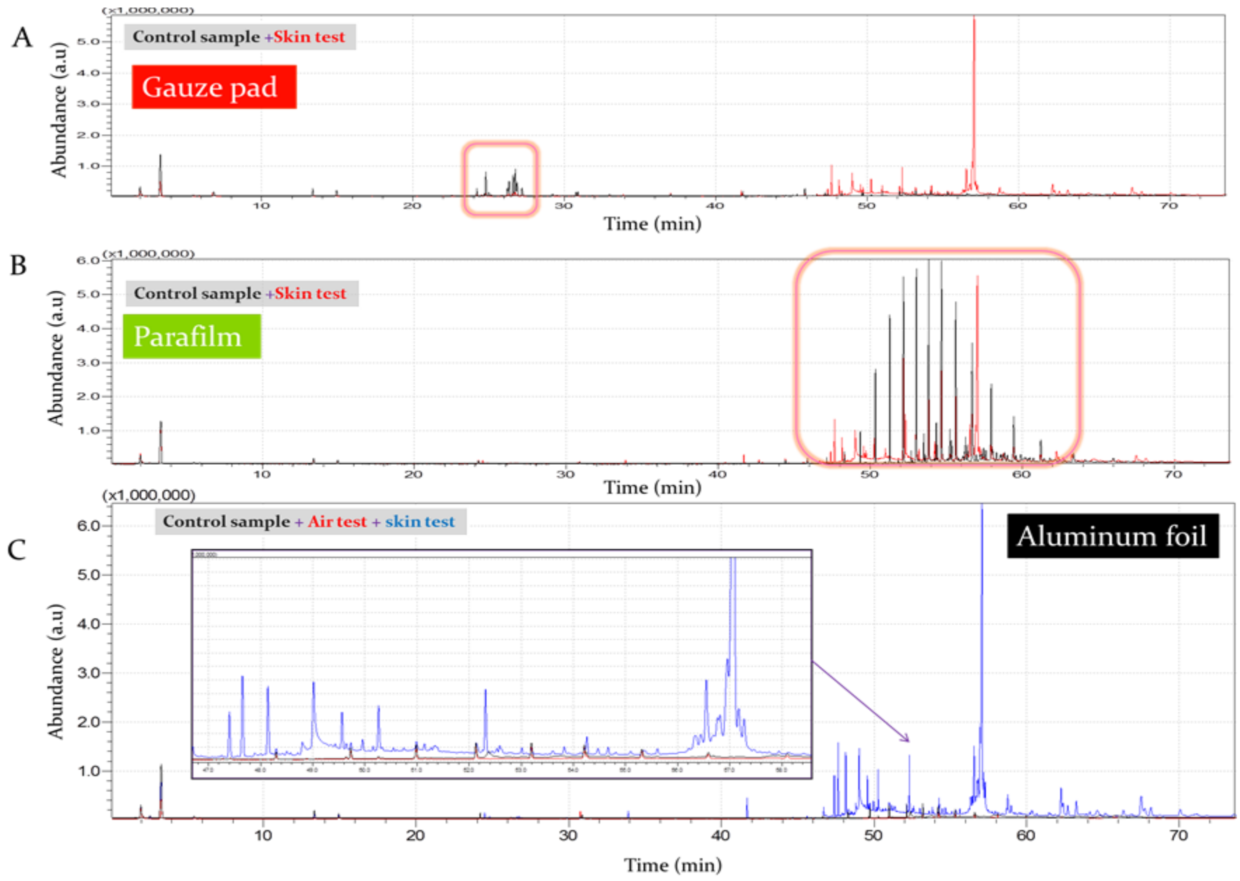

Figure S2. GC-MS chromatography for PDMS with different cover materials. a) Gauze pad; b) Parafilm and c) Aluminum foil. The insert is an enlargement of the shown area in the chromatography.

6. Sample duration from the skin was done for 60 min similarly to the literature. No further investigation was conducted as increasing the sampling time might cause refusal among potential participants.
7. As the distribution of secretion glands on the skin is heterogeneous, and therefore distinctive VOC profiles can be emitted from different parts of the body. As a result, we have sampled the skin in two regions including the bilateral inner arm (close to the armpit) and chest areas, as different VOC profiles are obtained. The results showed a significant difference in the VOC profile between the inner arm and chest (**Figure S3**). The skin cleaning method prior the sampling increased significantly the repetitiveness of the results and significantly reduced the differences between the different lateral sampling positions. Moreover, the percentage of the unique VOCs from the skin increased following the cleaning process. Skin cleaning process was tested in a serial experiment using distilled water, commercial alcohol preps and combination of both with different waiting time before sampling. It was found out that the optimal skin cleaning process was with alcohol prep 5 min before sampling. The average

percentage of relative standard deviations was lower for the series of experiments with the cleaning process (23%) compared to the experiment without cleaning process (59%).

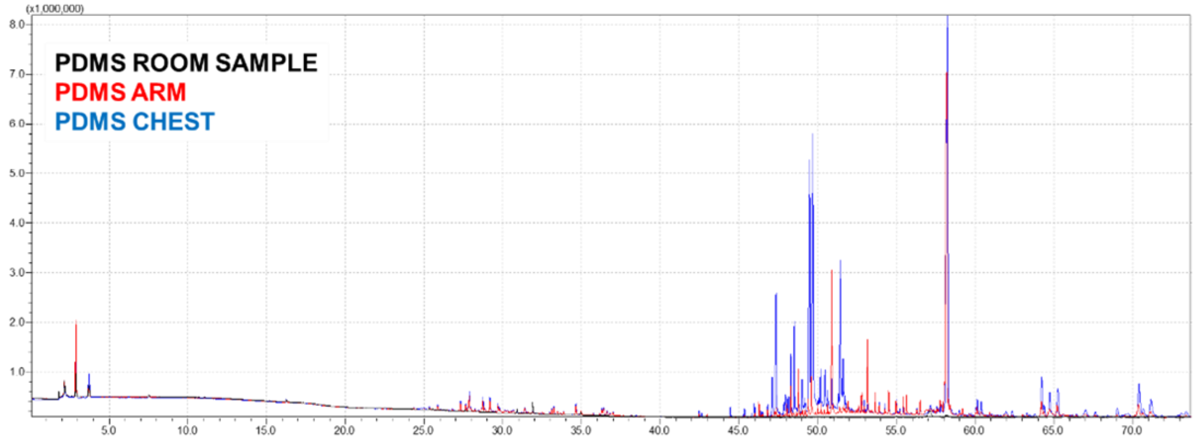

Figure S3. VOC profiles by PDMS absorbing material from the chest area (blue), inner arm area (red) and a room sample as a control.

2.2 Characterization of Tenax as a Sampling Tool

In order to increase the possibility to identify TB VOC profile via skin headspace, Tenax porous polymer (2,6-diphenyl-p-phenyleneoxide) (Buchem B.V.) as additional absorbing material was used. As PDMS and Tenax have different VOC absorbing materials, the use of both had the potential to cover a chemically wider range of emitted VOCs from the skin. Tenax is known as an excellent absorbing material for detection of VOCs via exhaled breath [71], once the Tenax is trapped inside a glass tube. In order to adjust the use of such a powdery polymer for skin sampling, several alternatives were investigated.

Initially, a commercial tag, 'ULTRA Passive Sampler for ppb-Level Organic Vapors' (www.skcinc.com) was used to trap the Tenax and allow it to be near the skin headspace without a direct contact with the skin that may cause irritation. As expected, the results showed VOC profiles from both inner arm and chest area, which were significantly different from the profiles obtained with the PDMS (*see* **Figure S4**). Since the solution of such tags were extremely expensive and required purchase of hundreds of tags for the clinical study, more cost affordable approaches were tested. The Tenax polymer was trapped in home-made envelopes sealed with heat presser which were made from polymeric membranes in order to allow vapor transfer, while ensuring no direct contact between the Tenax and the skin. The tested membranes included polyester with different meshes (30 and 47 µ) as well as 5 µ Polyethersulfone membrane (PES) that can control the preferred polarity of the transferred

gases for elimination of humidity effect. As can be seen from **Figure S5** and **Figure S6**, the skin VOC profiles obtained from Tenax with different envelops and Ultra tag are similar and there were no significant differences. As the PES membrane was hard to be sealed by heat, it was eliminated. 47µ polyester membrane was chosen due to high availability and low price.

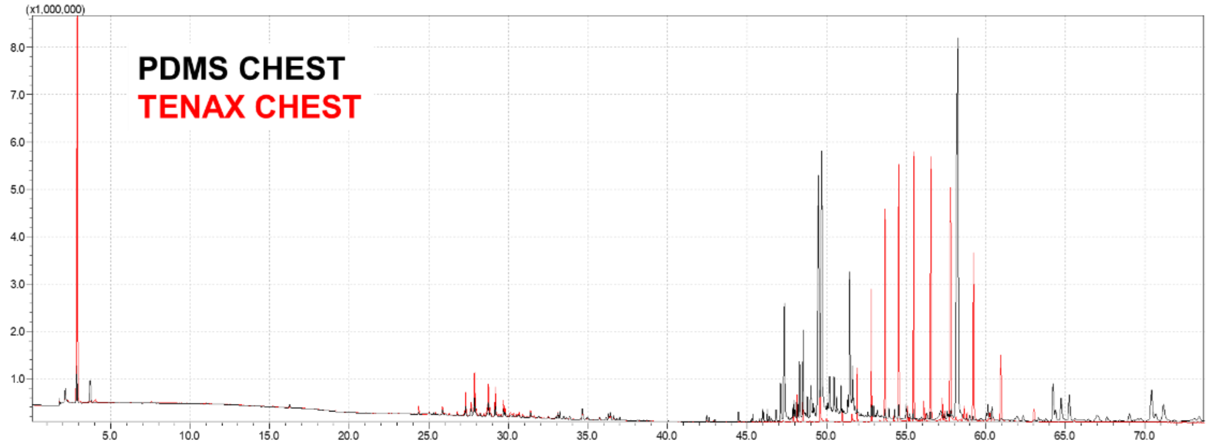

Figure S4. Differences in the skin VOC profile at the same body location, chest area, between the two absorbing materials, PDSM (black) and Tenax (red).

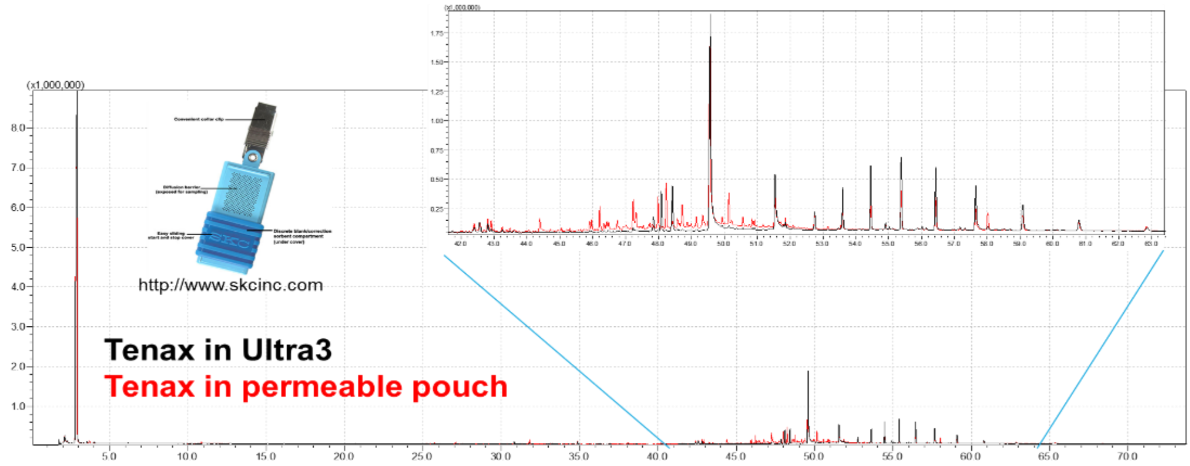

Figure S5. Skin VOC profiles extracted by Tenax polymer inside Ultra tag (black) and polymeric envelope (red).

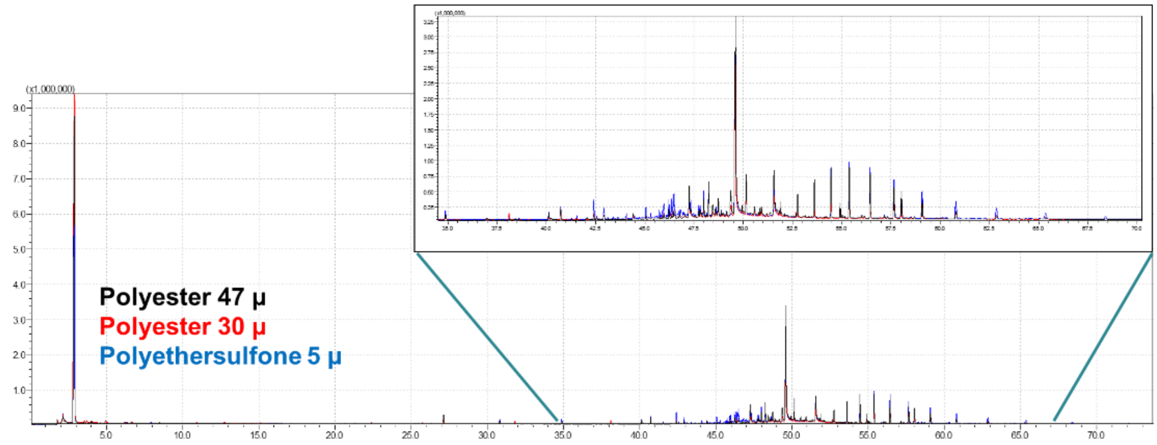

Figure S6. Skin VOC profiles extracted by Tenax polymer inside 47µ polyester membrane (black), 30 µ polyester membrane (red) and 5µ Polyethersulfone membrane.

The cleaning procedure of the polyester envelopes was evaluated and set to be the same as for the PDMS, expect for drying conditions of 100°C instead of 200°C. The Tenax polymer was chosen with the lowest available mesh (20/35), in order to ensure no direct contact with skin. 132±2 mg Tenax was thermally conditioned in a glass tube for the optimal duration of 3 hours at 300°C and 20 bar N_2 flow, after examining different combinations of time, temperature and pressure. Shelf-life examination was done at 4°C storage conditions. It was found that the samples in the glass vials, sealed with parafilm be stored up to 8 months, similarly to the PDMS.

2.3 Collection of Skin Headspace

Each volunteer was sampled as follows:

- Two Tenax patches on the inner arm area
- Two Tenax patches on the chest area
- Two PDMS patches on the inner arm area
- Two PDMS patches on the chest area
- One Tenax patch for room sampling, placed on a table during the skin measurement as a reference.
- One PDMS patch for room sampling, placed on a table during the skin measurement as a reference.

The duplicates of the different absorbing materials are used for two lab instruments:

- GC-MS for detection and characterization the skin TB-VOCs; and

- Laboratory nanomaterial-based sensors array chamber for sensor performance assessment.

3. DFA evaluation for preventing a potential overfitting

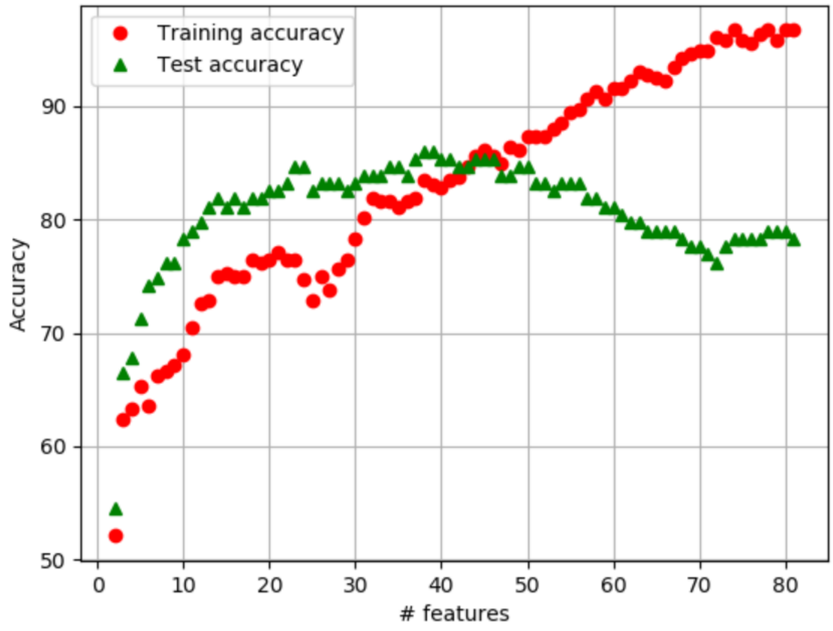

Figure S7. Developed DFA accuracies as a function of feature number for both training (70%) and test (30%) datasets.

4. Wearable device

The internet of medical things IoMT device consists of a Data Acquisition System coupled with the electrodes–Microchip to pick up vital signals from the human body. The device is well equipped with an analog front end which facilitates the signal extraction and conditioning. The system also has an analog to digital device (ADC), for converting the extracted analog signal to digital form, and a microcontroller which sends digital signals to the Bluetooth transceiver, which enables communication with external devices, such as: android mobile devices, computer Bluetooth, etc. Furthermore, this device contains USB-HID capabilities that enable communication with computer hardware, if needed, and functions as a battery charger interface.

Data Acquisition part is the most significant in the system. It includes sensors that transform a stimulus into an electrical signal that can then be converted by an ADC into a digital signal for processing. In addition, these systems often need a way to adjust different parameters of their sensors (gain, offset, *etc*). A complete data acquisition solution should be able to address this sub-system as well as the analog to digital conversion of the signals. One

of the most important building blocks in the data acquisition is the microcontroller unit MCU that is responsible of interconnecting the Bluetooth, ADC and the sensor. The following are the MCU features:

(i) Non-volatile Program and Data Memories:

- 16K - 128KBytes of In-System Self-Programmable Flash.
- 4K - 8KBytes Boot Code Section with Independent Lock Bits.
- 1K - 2KBytes EEPROM.
- 2K - 8KBytes Internal SRAM.

(ii) Peripheral Features:

- Four-channel DMA Controller.
- Eight-channel Event System.
- Five 16-bit Timer/Counters.
  Three Timer/Counters with 4 Output Compare or Input Capture channels.
  Two Timer/Counters with 2 Output Compare or Input Capture channels.
  High-Resolution Extensions on all Timer/Counters.
  Advanced Waveform Extension on one Timer/Counter.
- One USB device Interface.
  USB 2.0 full speed (12Mbps) and low speed (1.5Mbps) device compliant.
  32 Endpoints with full configuration flexibility.
- Five USARTs with IrDA support for one USART.
- Two Two-Wire Interfaces with dual address match (I2C and SMBus compatible).
- Two Serial Peripheral Interfaces (SPIs).
- AES and DES Crypto Engine.
- CRC-16 (CRC-CCITT) and CRC-32 (IEEE 802.3) Generator.
- 16-bit Real Time Counter with Separate Oscillator.
- One Twelve-channel, 12-bit, 2MSPS Analog to Digital Converter.
- One Two-channel, 12-bit, 1MSPS Digital to Analog Converter.
- Two Analog Comparators with Window compare function, and current source feature.
- External Interrupts on all General Purpose I/O pins.

- Programmable Watchdog Timer with Separate On-chip Ultra Low Power Oscillator.
- QTouch® library support Capacitive touch buttons, sliders and wheels Up to 64 sense channels.

(iii) Special Microcontroller Features

- Power-on Reset and Programmable Brown-out Detection.
- Internal and External Clock Options with PLL and Prescaler.
- Programmable Multi-level Interrupt Controller.
- Five Sleep Modes.
- Programming and Debug Interfaces PDI (Program and Debug Interface).

(iv) I/O and Packages

- 34 Programmable I/O Pins.
- 44 - lead TQFP.
- 44 - pad VQFN/QFN.
- 49 - ball VFBGA.

(v) Operating Voltage:

- 1.6 – 3.6V.

(vi) Operating Frequency

- 0 – 12MHz from 1.6V.
- 0 – 32MHz from 2.7V.

5. References

[1] S. Riazanskaia, G. Blackburn, M. Harker, D. Taylor, C. L. Thomas, *Analyst* **2008**, *133*, 1020.

[2] M. K. Nakhleh, H. Amal, R. Jeries, Y. Y. Broza, M. Aboud, A. Gharra, H. Ivgi, S. Khatib, S. Badarneh, L. Har-Shai, L. Glass-Marmor, I. Lejbkowicz, A. Miller, S. Badarny, R. Winer, J. Finberg, S. Cohen-Kaminsky, F. Perros, D. Montani, B. Girerd, G. Garcia, G. Simonneau, F. Nakhoul, S. Baram, R. Salim, M. Hakim, M. Gruber, O. Ronen, T. Marshak, I. Doweck, O. Nativ, Z. Bahouth, D. -y. Shi, W. Zhang, Q. -l. Hua, Y. -y. Pan, L. Tao, H. Liu, A. Karban, E. Koifman, T. Rainis, R. Skapars, A. Sivins, G. Ancans, I. Liepniece-Karele, I. Kikuste, I. Lasina, I. Tolmanis, D. Johnson, S. Z. Millstone, J. Fulton, J. W. Wells, L. H. Wilf, M. Humbert, M. Leja, N. Peled, H. Haick, *ACS Nano* **2017**, *11*, 112.
